# Medical knowledge representation enhancement in large language models through clinical tokens optimization

**DOI:** 10.1038/s41598-026-37438-6

**Published:** 2026-01-29

**Authors:** Qianqian Li, Jijun Tong, Shanna Liu, Chang Li, Jie Tang, Qingli Zhou

**Affiliations:** 1 School of Computer Science and Technology (School of Artificial Intelligence), Zhejiang Sci-tech University, Hangzhou, 310000 Zhejiang China; 2 School of Information Science and Engineering (School of Cyber Science and Technology), Zhejiang Sci-tech University, Hangzhou, 310000 Zhejiang China; 3 Department of Information Technology, International Institutes of Medicine, the Fourth Affiliated Hospital of School of Medicine and International School of Medicine, Zhejiang University, Yiwu, 322000 Zhejiang China

**Keywords:** clinical tokens, LLMs, tokenizer, natural language processing (NLP), Computer science, Software

## Abstract

During the training of medical large language models (LLMs), conventional tokenizers frequently segment domain-specific medical terms into multiple subword tokens, resulting in suboptimal recognition and representation of specialized vocabulary. As a consequence, the model encounters difficulties in effectively acquiring medical domain knowledge during the fine-tuning process. To address this limitation, the present study introduces “clinical tokens”—medical subword units—by augmenting the vocabulary of the original LLaMA2 tokenizer. This adapted tokenizer retains medical terms as whole tokens wherever feasible, thereby enhancing tokenization accuracy and enabling the model to learn and interpret medical knowledge more effectively. For downstream task adaptation, this study employs the Byte Pair Encoding (BPE) algorithm to construct a domain-specific vocabulary and tokenization model, ensuring the inclusion of medical subword units (clinical tokens). We compare the tokenization performance of three variants: the original LLaMA2 tokenizer, the Chinese-LLaMA2 tokenizer (expanded with an extended Chinese vocabulary), and the clinical token-augmented tokenizer. This was followed by fine-tuning the large language models on curated medical datasets. The experimental results indicate that the enhanced tokenizer improves encoding and decoding efficiency, extends the model’s effective context window, and yields superior performance on downstream medical tasks.

## Introduction

In natural language processing (NLP) tasks, text processing encompasses a range of applications, including machine translation^1^, sentiment analysis^2^, and automatic summarization^3^. Among these, text tokenization constitutes a foundational step. Most open-source large language models (LLMs) are pretrained predominantly on English corpora, resulting in a limited representation of Chinese tokens within their vocabularies. As a result, a single Chinese word often requires multiple tokens for representation, and entire phrases may be fragmented into even more tokens, hindering the model’s ability to capture specific semantic information. Chinese word segmentation (CWS) refers to the process of dividing Chinese text into discrete words or phrases. Unlike English and other languages that utilize whitespace as natural delimiters, Chinese text lacks explicit word boundaries. Consequently, different segmentation strategies applied to the same sentence can yield varying semantic interpretations, presenting a significant challenge for Chinese tokenization. To mitigate this issue, several studies have focused on expanding the original tokenizer’s vocabulary by incorporating additional Chinese tokens, thereby improving the rationality of segmentation and enhancing the model’s comprehension of Chinese text^21,22^.

With the advancement of LLMs, their applications in the medical field have expanded significantly, including automated medical documentation generation^4^ and clinical diagnosis^5^. In NLP tasks, input text must be tokenized into vector representations before being processed by the model. However, the specialized and domain-specific nature of medical texts presents a challenge for conventional tokenizers, which often fragment medical terminology into arbitrary subword units. This fragmentation leads to the loss of semantic integrity and negatively impacts performance on downstream tasks. In medical applications, accurately interpreting clinical data is essential for effective execution of downstream tasks. Precise tokenization of patient records significantly enhances LLMs’ ability to learn and comprehend medical knowledge. Traditional tokenization models—such as those used in LLaMA, Qwen, and ChatGPT—are trained primarily on English-language corpora. As a result, their vocabularies contain limited Chinese tokens and lack domain-specific medical lexicons, leading to suboptimal tokenization of medical data. To address this limitation, we employ the SentencePiece tokenizer^6^ to train a comprehensive vocabulary that encompasses a broad range of Chinese and medical terminology, thereby facilitating more accurate tokenization of clinical texts. This study introduces an innovative expansion of the LLaMA-2-7B model’s vocabulary by integrating Chinese medical tokens, thereby improving its capacity for domain-specific knowledge acquisition. Following this vocabulary augmentation, we perform continued pre–training and fine-tuning to develop Medical-LLaMA, a medical question answering (QA) model.

The main contributions of this paper can be summarized as follows:

1. We propose a lightweight domain adaptation method based on vocabulary expansion. In contrast to the prevalent heavy paradigms of large-scale continual domain-specific pre-training or adding adapter modules, our approach efficiently adapts the model from the representational source by injecting key domain-specific terms into the general-purpose model’s vocabulary, thereby maintaining higher computational efficiency and model generality.
2. We construct a hybrid evaluation framework that integrates automated metrics with LLMs assessment. Addressing the limitations of existing evaluation methods in terms of efficiency and depth, we systematically combine a semantic consistency metric (BERTScore) with multi-dimensional, fine-grained scoring from an LLM (DeepSeek-R1). This provides a new paradigm for generated text evaluation that balances scalability with in-depth analysis.
3. Experiments demonstrate that our model achieves significant and robust performance improvements on downstream tasks in the medical domain, accompanied by a substantial enhancement in its ability to capture the semantics of professional terminology.

## Related work

### Tokenization

To increase the accuracy of Chinese word segmentation (CWS), researchers have proposed various deep learning-based CWS models^7–9^, aiming to improve the model’s comprehension of Chinese text and thereby enhance performance in a range of text processing tasks. However, in specialized domains such as healthcare, law, and geoscience, even advanced CWS models often struggle to accurately capture domain-specific knowledge. These models frequently mis-segment technical terms, resulting in the distortion of their original semantic meanings. To overcome this limitation, scholars have undertaken domain-specific adaptations. For example, Chi Zhang^10^ developed a BERT-BiLSTM-CRF named entity recognition model tailored for criminal judgment documents. This model, when integrated with a custom dictionary and rule-based subsystems, constitutes a segmentation framework capable of accurately identifying and preserving specialized legal terminology, thereby significantly enhancing segmentation precision in legal texts. Similarly, recognizing that geological texts contain numerous unregistered domain-specific terms, Wei et al.^11^ proposed a Geoscience-Bidirectional Encoder Representations from Transformers (GeOBERT)-based CWS model specifically designed to handle various linguistic irregularities in the geoscientific literature. Guo et al.^12^ introduced BERTCWS, a purely unsupervised two-stage framework for domain-adaptive Chinese word segmentation. These efforts collectively address the challenges of geoscientific text segmentation by effectively decomposing texts into representative and semantically meaningful words or phrases, thereby achieving more accurate and contextually appropriate segmentation.

In NLP tasks, tokenization^13–15^ serves as a crucial preprocessing step, prompting the development of various tokenization algorithms such as WordPiece^16^, Byte Pair Encoding (BPE)^17^, and Unigram^18^. For languages lacking explicit word boundaries—such as Chinese and Japanese—WordPiece is comparatively less effective as a subword tokenizer. In contrast, BPE and Unigram have demonstrated superior performance in these linguistic contexts^19^. Yang et al.^20^ proposed the Less-is-Better (LiB) model, a novel tokenization approach designed for LLMs. The LiB model autonomously learns a comprehensive vocabulary encompassing subwords, full words, and multi-word expressions (MWEs). By merging frequently occurring adjacent subwords into longer units and pruning low-frequency or non-informative tokens, it effectively reduces both the total number of tokens and the overall vocabulary size. In a comparative study, Qarah and Alsanoosy^15^ pretrained multiple models via three tokenizers (WordPiece, SentencePiece, and Byte-Level BPE) across seven NLP tasks to analyze their differences. The results showed that the Byte-Level BPE (BBPE) tokenizer achieved remarkable efficiency, requiring the shortest pre–training time and fastest corpus tokenization speed. Additionally, it attains optimal compression rates, generating fewer tokens per input sequence, thereby enhancing processing speed and overall efficiency.

### Tokenizer vocabulary extension for LLMs optimization

In Chinese NLP tasks, segmenting Chinese characters into multiple tokens often hinders the efficiency of model training and inference. To mitigate this issue, Ji et al.^21^ expanded the LLaMA vocabulary and continued pre–training to enhance its processing capabilities for Chinese-language data. Similarly, Yiming Cui et al.[22] increased the original LLaMA vocabulary size to 49,953 tokens, significantly improving the model’s performance in Chinese comprehension and generation following pre–training and fine-tuning. In this study, we fine-tuned this Chinese-optimized model on medical QA data and compared its performance with that of our Medical-LLaMA model. Huang, Quzhe et al.^23^ adapted the English LLaMA model through continued pre–training and fine-tuning. Their pre–training strategy involved two stages. First, Chinese language capabilities were enhanced using multilingual corpora. This was followed by strengthening the model’s grasp of fundamental legal principles through the use of extensive raw legal texts, including legislative statutes, judicial interpretations, and official court records. For fine-tuning, they employed two data sources: (1) legal examination questions accompanied by ChatGPT-generated explanations and (2) synthetic legal consultation dialogs generated by ChatGPT. Xuanyu Zhang et al.[24] demonstrated notable success by jointly training BLOOM-176B on a mixed dataset comprising raw text and supervised fine-tuning data across general and financial domains. Their approach effectively facilitated knowledge injection and improved the model’s instruction-following ability.

## Tokenization algorithm

### BPE algorithm

BPE^17^ is a data compression algorithm designed to produce variable-length subword representations within a fixed-size vocabulary. The core mechanism of BPE involves iterative segmentation and statistical analysis of text. In each iteration, the algorithm identifies the most frequently co-occurring adjacent character or subword pair, merges them into a new symbol (or word unit), and replaces all instances of that pair throughout the text. This process continues until the desired vocabulary size is achieved or until all remaining character pairs occur only once. The final vocabulary retains both the original characters and the newly merged subwords. Owing to its simplicity and effectiveness, BPE has become the dominant subword tokenization strategy in contemporary NLP systems.

### BBPE algorithm

The BBPE algorithm operates at the byte level, employing the same iterative merging strategy as standard BPE. It begins by initializing the vocabulary with the 256 possible byte values defined by UTF-8 encoding, thereby ensuring broad compatibility across all languages. By treating each byte as a fundamental subword unit, BBPE effectively addresses the out-of-vocabulary (OOV) problem, particularly in languages such as Chinese and Japanese, where conventional tokenization methods often struggle with character segmentation and lexical coverage.

### Unigram algorithm

Unigram^18^ is a probabilistic subword segmentation algorithm widely employed in NLP tasks. In contrast to traditional BPE algorithms, Unigram adopts a probability model-driven approach that represents each word as a sequence of subword units and seeks to optimize segmentation by maximizing the overall likelihood of a sentence. The algorithm leverages the expectation-maximization (EM) technique to refine its vocabulary iteratively, preserving high-probability subword candidates while eliminating low-probability candidates at each optimization step. This probabilistic framework allows Unigram to support multiple plausible segmentation outcomes, making it particularly effective for morphologically rich languages and for handling OOV words.

### SentencePiece tokenization tool

SentencePiece^6^ is an unsupervised text tokenizer and detokenizer specifically developed for neural network-based text generation systems, where the vocabulary size is fixed before model training. It supports multiple subword segmentation algorithms, including BPE and unigram language modeling. A key innovation of SentencePiece lies in its ability to train subword models directly from raw text, eliminating the need for pre–tokenized input and thereby enabling consistent, language-agnostic tokenization across diverse corpora.

## Methods

### Data collection and preprocessing

This study compiled medical domain data for pre–training (Table 1) and expanded the Chinese medical vocabulary, as well as a curated subset of the general-domain SkyPile dataset^27^, at a 4:1 ratio. SkyPile is a large-scale, high-quality Chinese dataset specifically constructed for pre–training LLMs. It is derived from an extensive collection of publicly available Chinese web pages and subjected to comprehensive filtering processes, including aggressive deduplication and the sanitization of sensitive data. Tools such as fastText and BERT were employed to further eliminate low-quality content^28^. In addition, the study collected domain-specific medical QA data fine-tuned for vertical applications and high-quality general QA pairs generated by GPT-4, with a ratio of 6:5 between the two datasets. The medical QA data comprised open-source QA data from six clinical departments (Internal Medicine, Gynecology and Obstetrics, Surgery, Andrology, Pediatrics, and Oncology), with representative examples illustrated in Table 2, combined with the Shennong Traditional Chinese Medicine QA dataset at a 5:1 ratio. The dataset from six clinical departments comprises question-and-answer data from doctor-patient interactions, covering a range of medical topics, including disease diagnosis, medication guidance, and daily health consultations. All medical data are publicly accessible and have been de-identified. The GPT-4 QA pairs utilized in this research were sourced from the open-data GitHub project “Instruction-Tuning-with-GPT-4/GPT-4-LLM”. This project initially employed ChatGPT to translate 52,000 English instructions from Stanford’s Alpaca model into Chinese, subsequently requiring GPT-4 (whereas Alpaca used GPT-3.5) to generate responses in Chinese. Research has demonstrated that GPT-4 generates responses of superior quality, characterized by richer lexical diversity and longer sequences. This study subsequently conducted corresponding preprocessing on the medical QA data, removed incomplete and low-quality invalid data, and organized the remaining medical data into the required format for fine-tuning. For the QA data generated by GPT-4, checks were conducted on its completeness, repetitiveness, and content quality (where content quality was evaluated based on relevance, fluency, and completeness), and low-quality data was removed. Finally, the two types of data were uniformly blended to ensure the diversity of the training dataset.

**Table 1:** Pre-training medical dataset.

File name	Data details
CPT_tcmKnowledge_source1_17921	The dataset comprises 17,921 rigorously curated structured entries extracted from the authoritative “China Traditional Chinese Medicine Information Query Platform” database. This comprehensive collection spans specialized domains including diseases, symptoms, medical cosmetology, pharmaceuticals, Chinese medicinal herbs, health supplements, herbal formulations, medicinal diets, acupuncture points, and professional terminologies. Each entry has undergone meticulous manual verification by domain experts to ensure clinical accuracy and terminological precision.
CPT_tcmKnowledge_source2_12889	This dataset comprises 12,889 professionally curated medical entries containing detailed explanations of diseases, symptoms, and specialized terminology from authoritative sources including but not limited to ICD-10 and Chinese Medicine National Standards. The collection extensively covers key Traditional Chinese Medicine concepts such as herbal formulations, medicinal materials, acupuncture point combinations, and other essential TCM terms. All content has undergone rigorous manual editing and verification by qualified medical professionals to ensure the highest standards of accuracy and clinical validity.
CPT_medicalRecord_source1_61486	The dataset contains 61,486 clinical inquiry records from high-level licensed TCM physicians, consisting of high-quality natural language medical cases manually edited. Each record includes essential prescription elements such as patient inquiry guidance, symptoms, chief complaints, diagnoses, herbal formulas, and prescribed Chinese medicines.
CPT_medicalRecord_source2_15307	The dataset contains 15,127 clinical inquiry records from a renowned professor, featuring high-quality natural language medical cases that include essential prescription components such as patient inquiry guidance, symptoms, chief complaints, diagnoses, herbal formulas, and prescribed Chinese medicines, all meticulously edited by hand.
CPT_medicalRecord_source4_48665	The dataset comprises 48,665 clinical consultation records from a distinguished professor, consisting of high-quality natural language medical cases that include essential prescription components such as patient inquiry guidance, symptoms, chief complaints, diagnoses, syndrome patterns, herbal formulations, and Chinese medicinal ingredients, all meticulously edited by hand.

**Table 2:** Clinical data examples by department.

Department	Question	Answer
Internal Medicine	I have hypertension. My son-in-law brought some Codonopsis pilosula (Dangshen) for me to prepare as herbal tea during his recent visit. May I inquire whether it is safe for individuals with hypertension to consume Codonopsis pilosula?	Patients with hypertension may orally administer Codonopsis pilosula (Dangshen). Current research indicates that this herbal medicine exhibits lipid-lowering and hypotensive properties, while facilitating the clearance of metabolic waste products from the bloodstream. These mechanisms contribute to its potential stabilizing and preventive effects against coronary heart disease and other cardiovascular conditions. Regular consumption of Codonopsis pilosula may therefore help mitigate risks associated with the “three highs” (hypertension, hyperlipidemia, and hyperglycemia). Furthermore, beyond its cardiovascular benefits, this herb demonstrates additional pharmacological activities including tonifying effects and blood nourishment, central nervous system modulation, digestive function regulation, as well as splenic fortification and pulmonary reinforcement. Thank you for your consultation. We hope this evidence-based explanation proves beneficial for your health management.
Internal Medicine	Doctor, my uncle was hospitalized today. When I visited him at the hospital, I noticed the cardiovascular and cerebrovascular department was exceptionally crowded. Does cold weather disproportionately affect this specific patient population? May I inquire about the clinical severity of hypertension in elderly patients and whether it poses life-threatening risks?	Hypertension in elderly patients poses significant clinical risks, with documented multiorgan impact particularly affecting the cardiac, cerebral, and renal systems. Pathologically, it may trigger cerebral hemorrhage or cerebral infarction, potentially leading to critical outcomes including life-threatening conditions and hemiplegia. Cardiologically, it can induce myocardial hypertrophy progressing to cardiac insufficiency, while simultaneously elevating the risk of ischemic heart disease. Chronically, sustained hypertension accelerates renal arteriosclerosis, ultimately predisposing patients to renal insufficiency.

### Tokenizer training

**Fig. 1:**
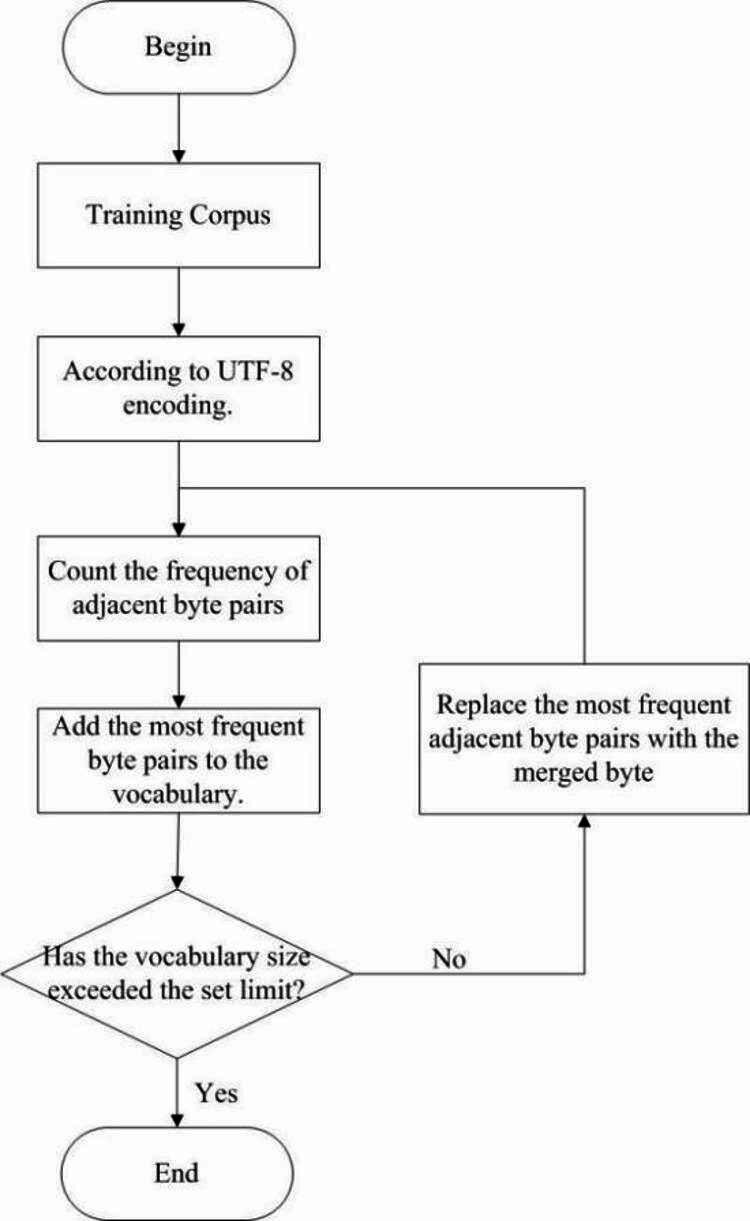
BBPE training workflow.

Due to the limited Chinese language coverage in the original LLaMA2 model, which includes a relatively small number of Chinese tokens, we expanded its vocabulary to incorporate Chinese medical terms, thereby enhancing the model’s capacity to learn both the Chinese language and domain-specific medical knowledge. Medical data from the pre–training corpus were selected as training material, and a BBPE SentencePiece tokenizer was trained on this corpus, resulting in a 12,000-token vocabulary. This vocabulary size was determined through iterative tokenizer training experiments, with 12k tokens yielding optimal performance in both model training and fine-tuning. Using the SentencePiece toolkit, we loaded and parsed both the newly trained tokenizer and the original LLaMA2 tokenizer, subsequently merging the new vocabulary with that of the original model. Duplicate tokens from the LLaMA2 vocabulary were removed, resulting in a final vocabulary size of 42,780 tokens. The tokenizer training process is depicted in Fig. 1.

For English and other Latin-based languages, BPE tokenization effectively mitigates OOV issues while maintaining a manageable vocabulary size^26^. However, in languages such as Chinese and Japanese, infrequent characters can unnecessarily inflate the vocabulary. To address this, BBPE offers a viable solution for handling OOV problems in Chinese. In practice, this can be implemented using SentencePiece by enabling the byte_fallback option (i.e., byte_fallback = true), which emulates the functionality of BBPE. Traditional BPE tokenizers may directly tag rare characters (such as uncommon Chinese characters) as [UNK], leading to information loss. However, all the characters are stored as bytes in computers (e.g., UTF-8 encoding). BBPE leverages this characteristic by employing Byte Fallback to ensure comprehensive coverage. Byte Fallback is a fault-tolerant strategy in computer science (particularly in text encoding and natural language processing) designed to handle characters unrecognized by the current character encoding system. Its core principle is that when encountering an undecodable character, the system automatically converts it to a raw byte sequence or a predefined substitute representation rather than directly throwing an error or losing data, thereby enabling byte-based byte pair encoding. For tokenizer training, the parameters were configured as follows: model_type was set to BPE, vocab_size was set to 12,000, and byte_fallback was set to true. This configuration yielded a tokenizer model and vocabulary capable of capturing high-granularity medical terms, thereby enhancing the model’s ability to learn N-gram features.

To determine the most effective tokenizer configuration, the study explored five distinct combinations of training parameters. These included vocabulary sizes of 32,000, 22,000, and 12,000; character coverage values of 1 and 0.9995; and normalization rules of either NFKC or NMT-NFKC. All other training parameters were held constant. Multiple tokenizers were trained under these configurations, and the LLaMA2 model was subsequently subjected to continued pre–training and fine-tuning using each tokenizer. The influence of these parameters on both tokenizer performance and downstream model outcomes was systematically evaluated, resulting in the selection of a configuration that achieved the most favorable results. The evaluation results were primarily determined by DeepSeek-R1’s model reasoning scores and BERTScore metrics, as detailed in Sect. Results and analysis 

### Embedding layer initialization

After the vocabulary is merged, the embedding layer is adjusted to accommodate the new vocabulary size. Specifically, its shape was modified to new_vocab_size × dim and re-initialized accordingly. The vector representations of existing tokens from the original vocabulary remain unchanged. In contrast, two distinct strategies—mean imputation and random sampling—are employed to initialize the vectors for newly added tokens. During this process, the embedding layer of the original LLaMA2 model was replaced, and an identical initialization operation was applied to the lm_head layer. To compare the efficacy of these initialization approaches, this study conducted controlled experiments under identical training and evaluation configurations. The performance of both methods was ultimately assessed based on the BERTScore of model-generated outputs, leading to the adoption of random sampling for initializing the embedding layer.

The mean imputation method involves decomposing each new token into known subword units using the original tokenizer and then computing the mean vector from the encoded vectors of these subword units, which serves as the initial vector representation for the new token. The random sampling method employs a truncated normal distribution for initialization: values are sampled from a normal distribution with a mean of 0 and a standard deviation of 0.02, maintaining parameter distribution characteristics similar to those of the original pre–trained model and ensuring that the initial values of new tokens remain within a reasonable range.

### Continued pre-training and instruction fine-tuning

#### Continued pre-training

Following the initialization of the embedding and lm_head layers, the model underwent continued pre–training, primarily to facilitate adaptation to the newly introduced medical tokens. Ideally, the initial training phase would be limited to the embedding layer, allowing for the adjustment of medical word vectors while preserving the invariance of the original model. However, owing to computational constraints and the minimal performance gain observed in preliminary experiments, this step was omitted. Instead, the study proceeded directly with full–model pre–training using Low–Rank Adaptation (LoRA) techniques, and the pre–training data were divided into training and test sets at a ratio of 10:1. The primary objective of this training was to refine the embedding layer, ensuring that the newly integrated Chinese medical token vectors were well aligned with LLaMA2’s pre–existing semantic structure. This strategy aimed to preserve the model’s original capabilities while enabling robust representation and understanding of Chinese medical terminology, thereby equipping the model with foundational domain-specific knowledge.

#### Instruction fine-tuning

After loading the medical QA dataset, the tokenizer segments the textual data into subword tokens, which are subsequently transformed into vector representations through the embedding layer. These vectors were then input into the model for fine-tuning using the LoRA approach. The training, validation, and test sets were partitioned in a ratio of 10:1:1, with the test set containing 12,000 instruction-response pairs. The training set is used for updating the model parameters, the validation set is used for evaluating the model’s loss, and the test set remains completely withheld throughout the entire training process, being used solely for a single, final evaluation of model performance. In this study, both the modified Medical-LLaMA model and the Chinese-LLaMA2 model^22^ were fine-tuned on an identical medical QA dataset. The sole distinction between the two fine-tuning processes lies in the tokenization step; all other training procedures and hyperparameters were held constant. During the fine-tuning phase, both models learned to generate responses based on their pre–existing knowledge in conjunction with the QA logic present in the training data. Next, inference was conducted on the test set to evaluate model performance. A systematic comparison of the outputs was conducted to evaluate the impact of tokenizer modifications on model behavior and accuracy. This experiment utilized 120,000 fine-tuning data samples and was trained for only one epoch, which fundamentally prevents data memorization. Furthermore, the LoRA fine-tuning architecture significantly constrains both the number of trainable parameters and the model capacity. Additionally, multiple regularization mechanisms (weight decay + dropout) were employed, consequently maintaining a minimal risk of overfitting.

In addition to the primary medical QA task, this study incorporates two downstream tasks—medical named entity recognition and medical text classification—to comprehensively evaluate the model’s generalization capability across diverse medical scenarios. Both tasks are conducted using the CBLUE benchmark. We adopt the same fine-tuning strategy as the medical QA task to ensure consistency in the training pipeline across different tasks. For the medical named entity recognition task, the model is required to identify and classify entities such as diseases, symptoms, and drugs in text, with performance measured using BERTScore to assess semantic alignment between generated sequences and reference annotations. The medical text classification task aims to categorize clinical trial eligibility criteria into predefined classes, with test set performance evaluated comprehensively from two dimensions: semantic matching (BERTScore) and classification consistency (F1-score). To maintain consistency in experimental setup, both tasks strictly adhere to the training, validation, and test set splits defined by the CBLUE benchmark.

### Model training details

The complete training pipeline consists of continued pre–training and instruction fine-tuning, both of which are implemented via LoRA. The experimental parameters for each phase are summarized in Table 3. Given the substantial scale of the pre–training dataset, a larger effective batch size (16 × 4) was employed to increase training throughput and stabilize the optimization process. As pre–training involves continuous adaptation to massive data distributions, the model weights require significant adjustments. Consequently, a relatively high learning rate (2 × 10^–4^) was adopted to facilitate efficient parameter space exploration and accelerate convergence. For instruction tuning, the dataset scale is considerably smaller than that of the pre-training data. A reduced effective batch size (8 × 4) was implemented to improve the generalization performance, optimize GPU memory utilization, and potentially promote convergence to superior local minima. Since instruction tuning builds upon a comprehensively pre–trained model with established capabilities, the objective focuses on behavioral refinement rather than fundamental architectural changes. Thus, a conservatively low learning rate (5 × 10^–5^) was applied to prevent catastrophic forgetting while ensuring stable convergence. The sequence length limit of 1024 tokens was selected as an optimal balance between contextual capacity and computational feasibility for 7B-scale models within conventional hardware constraints.

**Table 3:** Experiment parameters.

Experimental setup	Continued pre-training	Instruction fine-tuning (Medical-LLaMA-2-7b)	Instruction fine-tuning (Chinese-LLaMA-2-7b)
Batch Size	16	8	8
Gradient accumulation steps	4	4	4
Learning Rate	2e-4	5e-5	5e-5
Training Steps	1800	3750	3750
Max Length	1024	1024	1024
Trainable Parameters(%)	7.08%	0.29%	0. 30%
Training Device	80G*1	80G*1	80G*1

### Evaluation criteria

This study employs BERTScore^25^, an evaluation metric for machine translation and text generation that utilizes pretrained BERT models to compute the semantic similarity between generated and reference texts by measuring the cosine similarity of contextual embeddings. The BERTScore evaluates text quality in three dimensions: precision (P), which reflects the proportion of matching content in the generated text; recall (R), which indicates coverage of the reference content; and the F1 score (F), which represents the harmonic mean, which addresses the limitations of n-gram methods in capturing semantic relationships.

**Fig. 2:**
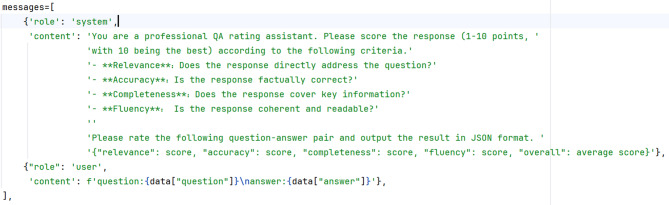
Prompt scoring template (Translated into English Version).

Furthermore, we implement Deepseek-R1’s scoring framework (prompt template shown in Fig. 2) to assess model-generated medical answers comprehensively across four criteria: relevance (question-directed focus), accuracy (factual correctness), completeness (key information inclusion), and fluency (linguistic coherence). Each response receives multidimensional ratings, with the final model performance determined by averaging scores across all test samples.

## Results and analysis

This study explored multiple configurations of tokenizer training parameters, followed by continued model pre–training and task-specific fine-tuning for each tokenizer variant. The performance of the resulting models was evaluated to determine the effectiveness of the different tokenizers, with the outcomes summarized in Table 4. The results indicate that the tokenizer performed optimally when the vocabulary size was set to 12,000, the character coverage was set to 1, and the text normalization rule was set to NFKC. The experiment further revealed that increasing the vocabulary size does not necessarily yield performance gains. While tokens with higher granularity allow the model to learn the semantics of frequently occurring, complete medical terms more effectively, an excessively large token can compromise semantic coherence between tokens and their constituent subwords. For example, “跑步机” (treadmill) and “跑步” (running) may be encoded as entirely separate tokens, thereby requiring the model to learn their meanings independently despite their clear semantic connection.

**Table 4:** Performance evaluation of models with different tokenizers.

Vocab-size(k)	32	32	22	12	32
Character-coverage(%)	0.9995	1	1	1	1
Normalization	nfkc	nfkc	nfkc	nfkc	nmt-nfkc
Relevance(1–10)	7.437	7.283	**7.658**	7.633	7.592
Accuracy(1–10)	6.720	6.716	6.808	**6.992**	6.792
Completeness(1–10)	5.976	5.900	6.108	**6.292**	6.075
Fluency(1–10)	8.927	8.916	8.916	**9.017**	8.625
Overall(1–10)	7.193	7.215	7.376	**7.495**	7.290
BERTScore(0–1)	0.729	0.727	0.726	**0.732**	0.723

significance of bold indicates the numerical values

In Tables 4, 5, and 7, values in bold denote the maximum value in each row, representing the best performance for that specific metric.

For the initialization of the model’s embedding layer, the Medical-LLaMA model using mean imputation achieved a BERTScore of 0.732 and a DeepSeek-R1 score of 7.260, whereas the model employing random sampling attained a BERTScore of 0.734 and a DeepSeek-R1 score of 7.425, outperforming the mean imputation approach. Theoretically, mean imputation should yield better performance, as it initializes new tokens with vectors closer to their semantic meanings. However, upon careful analysis, two primary reasons likely explain this discrepancy. First, the mean imputation method relies on a strong assumption that the semantics of new tokens are static, linear combinations of their sub-token meanings. While this may hold about for certain compound words (e.g., “男” + “人” → “男人”), it proves fragile for most new tokens—particularly specialized medical terms, jargon, or words representing entirely novel concepts. Starting from a biased and inaccurate semantic initialization, the model must first “unlearn” this incorrect representation before acquiring the correct semantics, thereby increasing the learning burden. Moreover, this method completely discards the sequential information of sub-tokens, which is crucial for semantic composition. In contrast, random initialization introduces no static semantic bias. It provides a blank slate, allowing the model to dynamically derive the semantics of new tokens entirely from their actual contextual usage in the training data, thereby capturing their true, holistic meanings more accurately. Second, mean imputation begins optimization from a biased starting point, potentially trapping the model in a local, suboptimal “basin”. The initial inaccurate semantics may act as an anchor, requiring gradient descent to exert greater effort to escape and move toward the truly optimal configuration. Random initialization, although it starts “blind”, avoids such anchoring effects, enabling the optimizer to more freely discover an embedding configuration better aligned with the global model dynamics. Consequently, the random sampling method demonstrated superior performance, and this study adopted it for initializing the embedding layer.

After expanding the vocabulary of the LlaMA-2-7B model, the average number of tokens required to represent a Chinese character decreased relative to the original tokenizer. Using the original LLaMA2 vocabulary, tokenizing a single Chinese character required an average of 1.52 tokens. In contrast, the revised vocabulary reduces this number to about 0.74 tokens, thereby enhancing both encoding and decoding efficiency. In the context of the same medical QA task, fine-tuning on 120,000 QA samples took 9 h and 18 min with the original LLaMA2 model. However, the vocabulary-expanded model completed the fine-tuning process in just 4 h and 42 min, indicating a marked improvement in training efficiency. This optimization notably reduces GPU resource consumption, particularly for larger-scale models and more extensive training datasets.

The inclusion of domain-specific terms such as “无明显诱因” (no apparent cause), “血常规” (routine blood test), and “糖尿病” (diabetes mellitus) in the vocabulary directly enhances the model’s effective context window. The original LLaMA2 model, with a 4,096-token context length and an average 1.52-token-per-character ratio, could process only ~ 2,695 Chinese characters. In contrast, Medical-LLaMA (with an optimized value of 0.74 tokens/character) achieves a 105% increase to 5,535 characters within the same token limit. This expansion significantly improves performance on downstream medical tasks by enabling richer contextual understanding.

The Medical-LLaMA model achieved a BERTScore of 0.732, representing a modest yet consistent improvement over the original LLaMA2 model (0.715) and Chinese-LLaMA2 (0.713)^22^, as detailed in Table 5. The inference results comparing Medical-LLaMA and Chinese-LLaMA2 are presented in Table 6. Although both models generally performed well in answering medical questions, Chinese-LLaMA2 displayed limitations in specific scenarios, whereas Medical-LLaMA consistently provided more accurate and contextually appropriate responses. For example, in Question 1 concerning tenesmus, a symptom of the digestive system, Chinese-LLaMA2 suggested traditional Chinese medicines without tailoring the recommendations to the patient’s condition. In contrast, Medical-LLaMA offered herbal treatments aligned with symptom-specific analysis. In Question 2, the Chinese-LLaMA2 did not directly address the patient’s inquiry; instead, it offered general advice to seek medical attention. Medical-LLaMA, however, responded with a clinically relevant and targeted answer. These findings underscore the benefit of incorporating domain-specific vocabulary, such as medical terminology, into the tokenizer. The inclusion of specialized tokens enhances the model’s comprehension of medical concepts and improves its ability to discern logical relationships during fine-tuning. As a result, the model is better equipped to generate precise, problem-specific outputs in specialized applications.

**Table 5:** Model BERTScore.

BERTScore(0–1)	Medical-LLaMA	Chinese-LLaMA[22]	LLaMA2
P	**0.731**	0.719	0.715
R	**0.735**	0.708	0.715
F1	**0.732**	0.713	0.715

significance of bold indicates the numerical values

**Table 6:** Model inference results.

Question	Chinese-LLaMA2 model responses	Medical-LLaMA model responses
I have symptoms of tenesmus (rectal urgency). Are there any Chinese herbal medicines you can recommend?	I am sorry to hear about your discomfort. Here are some commonly used Chinese herbal medicines that may help: \n\n1. Ginseng – Replenishes qi and blood, nourishes vitality. Suitable for weakness and fatigue caused by qi and blood deficiency.\n\n2. Astragalus – Strengthens qi, boosts immunity. Often used for general debility or frequent illnesses like colds.\n\n3. Chinese Angelica Root – Promotes blood circulation, regulates menstruation, and relieves pain. Commonly used for menstrual disorders or cramps.\n\n4. Rehmannia – Nourishes the kidneys, strengthens bones. Helps with symptoms like lower back pain or dizziness due to kidney yang deficiency.\n\nImportant Note: These are general suggestions. Herbal remedies should be tailored to your specific constitution and condition. Please consult a licensed Traditional Chinese Medicine (TCM) practitioner or doctor before use.	Tenesmus (rectal urgency) is a common digestive disorder, and Chinese herbal medicine can help alleviate its symptoms. Here are some frequently used herbs: \n\n1. Coptis – Clears heat, detoxifies, and dries dampness to relieve diarrhea or dysentery.\n\n2. Costus Root – Regulates qi, resolves dampness, and relieves pain. Used for intestinal cramps or abdominal discomfort.\n\n3. Alisma – Promotes urination, reduces swelling, and stops diarrhea. Effective for edema or watery stools.\n\n4. Licorice – Harmonizes herbal formulas, eases spasms, and reduces pain. Often added for gastrointestinal inflammation.\n\nImportant Notes: These suggestions are for reference only. Always consult a TCM practitioner or doctor for personalized treatment.Seek immediate medical care if symptoms persist or worsen.
I have numbness in my limbs. What should I do? Can you recommend any Chinese herbal medicines or formulas?	I am sorry to hear about your discomfort. Based on the information you have provided, I strongly recommend seeking prompt medical attention for proper diagnosis and treatment by a qualified doctor. Chinese herbal medicines or formulas should be tailored to individual conditions, so it’s best to use them under professional medical guidance. Wishing you a speedy recovery!	Numbness in the limbs may be caused by insufficient qi and blood, insufficient liver and kidneys, or other factors. Here are some commonly used Chinese medicines and prescriptions: \n\n1. Angelica blood soup: composed of angelica, cooked ground, white peony, chuanxiong, cinnamon twig, and ginger, which has the effects of nourishing blood and nourishing blood, warming the meridians and dissipating cold.\n\n2. Siwu soup: composed of angelica, rehmannia rehmannia, white peony, and chuanxiong, which has the effects of nourishing blood and nourishing blood, regulating menstruation and relieving pain. \n\n3. Ginseng Astragalus Soup: Composed of ginseng, astragalus, atractylodes, poria, and licorice, it has the effects of nourishing qi and yang, strengthening the spleen and appetizing.\n\n4. Bazhen Soup: Composed of ginseng, astragalus, atractylodes, poria, roasted licorice, jujube, wolfberry, and angelica, it has the effect of nourishing qi and blood and regulating the spleen and stomach.\n\nThe above prescriptions need to be used under the guidance of a doctor, and it is recommended that you go to a regular hospital or traditional Chinese medicine clinic for detailed diagnosis and treatment plan.

The test data were used as input questions for inference via the Medical-LLaMA, Chinese-LLaMA2, and original LLaMA models, with their outputs serving as answers. The Deepseek-R1 model then evaluated these responses across multiple metrics, and the detailed results are presented in Table 7. Compared with both the original LLaMA2 and the Chinese-LLaMA2 models, the Medical-LLaMA model demonstrated superior scores across all the evaluation dimensions. This outcome indicates that Medical-LLaMA achieves enhanced performance after fine-tuning, resulting in more precise comprehension of medical knowledge and improved accuracy in addressing clinical questions. The comparative results substantiate that domain-specific vocabulary optimization and medical-focused training significantly enhance model capabilities for medical domain applications. Furthermore, we conducted a Wilcoxon one-tailed test on the DeepSeek-R1 scores of the Medical-LLaMA and Chinese-LLaMA models. The results revealed a test statistic of 262.0, p value of 0.01374, and a Cliff’s Delta effect size (r) of 0.532. The one-tailed test reached statistical significance at the 0.0137 level (i.e., *p* < 0.05), allowing rejection of the null hypothesis (no difference between the two groups) at the α = 0.05 significance level. This finding indicates a statistically significant difference with a clear directional tendency—consistent with the hypothesis that the Medical-LLaMA group significantly outperforms the Chinese-LLaMA group. A Cliff’s Delta value of 0.532 corresponds to a large effect size, suggesting low overlap and a pronounced difference between the two groups. To validate the general domain performance of the Medical-LLaMA model, this study introduces the instruction-tuned LLaMA2-Chat model as a baseline for comparison with Medical-LLaMA. The experimental results demonstrate that Medical-LLaMA’s comprehensive performance in general domains not only shows no degradation but even slightly surpasses that of the baseline model. This finding empirically validates that the domain adaptation method employed in this study effectively enhances the capabilities of medical professionals, while maintaining performance on general tasks.

**Table 7:** Deepseek-R1 evaluation scores.

Deepseek-R1 score(1–10)	Medical-LLaMA	Chinese-LLaMA2^22^	LLaMA2
relevance	**7.633**	7.286	6.899
accuracy	**6.992**	6.254	5.286
completeness	**6.292**	5.741	5.323
fluency	**9.017**	8.611	7.566
overall	**7.495**	6.982	6.275

significance of bold indicates the numerical values

This study supplemented experiments with two tasks: medical named entity recognition and medical text classification, to validate the effectiveness of vocabulary expansion. In the medical entity recognition task, the Chinese-Llama model with Chinese expansion achieved a BERTScore of 0.949, while the further optimized Medical-Llama model reached 0.954 on this metric, marginally outperforming the former. In the medical text classification task, the model with expanded medical vocabulary achieved a macro-F1 score increase of 0.061 in domain-specific entity recognition compared to the Chinese-Llama model, along with a higher BERTScore than the baseline model. These results collectively demonstrate that the vocabulary expansion strategy delivers consistent performance gains across different types of medical natural language processing tasks.

## Conclusion

For training LLMs in vertical domains, expanding the vocabulary with domain-specific terms enhances the model’s ability to acquire and comprehend specialized knowledge, thereby facilitating more effective fine-tuning and improved performance on downstream tasks. Compared to the original LLaMA2 tokenizer and Chinese-LLaMA2, which expands the Chinese vocabulary, our medical-domain tokenizer incorporates clinically relevant subword units, referred to as clinical tokens. These tokens possess greater granularity, enabling the capture of nuanced semantic meanings while reducing the overall number of tokens required for text representation, thereby improving computational efficiency. Moreover, within a fixed context window, the increased character coverage per token allows the model to process longer textual sequences, enhancing both understanding and generative capabilities. Together, these optimizations contribute to accelerated training and inference times, while significantly enhancing the model’s proficiency in acquiring medical knowledge.

This study has several limitations, which also point to worthwhile directions for future exploration. First, at the model and training level, this research is based solely on the LLaMA-2-7B model with medical vocabulary expansion and employs parameter-efficient fine-tuning methods such as LoRA. Although significant performance improvements were achieved on the target medical tasks, constrained by computational resources, validation on larger-scale models (e.g., 13B or 70B) or models with different architectures was not conducted. Consequently, the broad generalizability of the conclusions remains to be verified. Furthermore, while efficient fine-tuning methods substantially reduce computational costs, their performance gains may theoretically remain below the upper bound achievable by full-parameter fine-tuning, especially in complex scenarios requiring deep and comprehensive updates to the model’s internal knowledge representations, where the extent of improvement may be relatively limited. Second, regarding evaluation, the effectiveness of our proposed “DeepSeek-R1 + BERTScore” hybrid assessment framework has been validated on structured, semantically clear QA tasks. However, for tasks that highly depend on creative thinking, domain-specific insight, or complex clinical reasoning, automated scoring systems may struggle to fully capture the core dimensions and nuanced differences in quality. The ultimate assessment of such tasks still requires integration with qualitative judgment and in-depth analysis by domain experts.

Despite the aforementioned limitations, this study provides a feasible pathway for achieving domain adaptation under constrained resources and provides a reproducible reference framework for automated assessment. Future work could delve deeper into directions such as scaling to larger models, conducting full-parameter fine-tuning, and exploring human-AI collaborative evaluation.

## Data Availability

The datasets supporting the findings of this study are publicly available on GitHub and Hugging Face. Specifically:1. Medical domain pre-training data: Available at SylvanL/Traditional-Chinese-Medicine-Dataset-Pretrain on Hugging Face.2. General-domain pre-training data (SkyPile dataset): Available at Skywork/SkyPile-150B on Hugging Face.3. Medical QA data: Available at Toyhom/Chinese-medical-dialogue-data on GitHub and michaelwzhu/ShenNong\_TCM\_Dataset on Hugging Face.4. General-purpose GPT-4 generated QA pairs: Available at Instruction-Tuning-with-GPT-4/GPT-4-LLM on GitHub.
